# Invisible, Uncontrollable, Unpredictable: Illness Experiences in Women with Sjögren Syndrome

**DOI:** 10.3390/jcm13113228

**Published:** 2024-05-30

**Authors:** Andrea Herrera, Loreto Leiva, Iris Espinoza, Matías Ríos-Erazo, Nailah Shakhtur, Pamela Wurmann, Gonzalo Rojas-Alcayaga

**Affiliations:** 1 Behavioral Science Area, Institute for Research in Dental Science, Faculty of Dentistry, Universidad de Chile, Santiago 8380544, Chile; aherrera@odontologia.uchile.cl (A.H.); gorojas@odontologia.uchile.cl (G.R.-A.); 2 Department of Psychology, Faculty of Social Sciences, Universidad de Chile, Santiago 7800284, Chile; loretoleivab@u.uchile.cl; 3 Department of Oral Medicine and Pathology, Faculty of Dentistry, Universidad de Chile, Santiago 8380544, Chile; iespinoza@odontologia.uchile.cl; 4 National Association of Sjögren Patients of Chile, Santiago 8320214, Chile; nshakhtur@gmail.com; 5 Medicine Department, Dental and Maxillofacial Service, Clinical Hospital, Universidad de Chile, Santiago 8380456, Chile; loretopwurmann@hcuch.cl

**Keywords:** Sjögren Syndrome, qualitative research, grounded theory, illness experience

## Abstract

**Background/Objectives**: Sjögren’s Syndrome (SS) is a chronic degenerative rheumatic disease. Because of its chronic nature, it significantly affects the quality of life of those who suffer from it. **Methods**: This qualitative study investigated disease experience among women suffering from SS to understand its impact on their overall well-being. In-depth interviews were conducted with 15 women who suffer from SS. Interviews were analyzed using the Grounded Theory methodology, using open, axial, and selective coding. **Results**: Three central phenomena of disease experience were identified: invisibility; uncontrollability; and unpredictability. **Conclusions**: SS disease experience has a strong imprint on emotional well-being and sense of self-control among middle-aged women. Understanding SS impacts on women’s lives is important to better understand the disease and contribute to recognizing potential areas of management and social support in relevant windows of opportunity within the health-disease continuum.

## 1. Introduction

Sjögren’s Syndrome (SS) is a chronic autoimmune disorder [1] within the rheumatic diseases and conditions’ spectrum. It shares common biological and psychosocial characteristics with many other chronic diseases (CD), including impaired physical performance, high prevalence of fatigue, depression, and anxiety [1]. SS affects approximately 0.1% of the global population, with prevalence rates ranging from 0.02 to 0.6% [2,3,4]. It is more commonly observed in women over the age of 55 [5,6].

Clinical manifestations of SS are diverse, with both silent and symptomatic phases occurring concurrently. The Hallmark signs include oral and ocular dryness [7], affecting nearly half of all patients [8]. Dryness can extend to other mucosal surfaces, including airways, the digestive tract, and the vagina [1,9]. A lower salivary production may cause dysphagia, dysgeusia, pain, and burning sensation [1]. The mouth may reveal dry, erythematous oral mucosa, a lobulated or depapillated tongue, dental caries, periodontal disease, bacterial sialadenitis, *Candida* infection, and angular cheilitis [7,10]. Glandular swelling may also be present, affecting the parotids, submandibular, or sublingual glands [11]. Respiratory tract dryness can lead to persistent hoarseness and a chronic, non-productive cough [12]. Skin involvement is characterized by cutaneous xerosis, while reduced vaginal secretion can result in dyspareunia and local discomfort [7,13]. Additionally, impaired exocrine gland function in the digestive tract may affect pancreatic dysfunction and hypochlorhydria [14]. In primary SS patients, chronic tubulointerstitial nephritis is prevalent, which clinically translates into renal tubular acidosis [15]. The latter may induce renal calculi and osteomalacia [16,17].

Like other CDs, it affects people’s psychological well-being and quality of life. They typically become a problem that affects people’s functional lives, limiting their employment, educational, and social possibilities [18]. Physical and mental fatigue symptoms, as well as body pain, are frequent and limit daily activities [19,20]. The dryness of the vagina and vulva interferes with sexual activity and desire [21]. Chronicity is difficult to deal with; due to fatigue, physical pain, and anxiety as a result of chronic conditions, the mechanisms to cope with them are also limited. Treatment for SS aims to control, alleviate symptoms, and prevent complications, as it is impossible to halt disease progression in the majority of patients [18,22,23].

People with CD, such as SS, experience a great number of life-disrupting changes, and these are only accessible by exploring the interpretations of the individual attributes of the change. Then, the “patient’s point of view” takes on special relevance because it emphasizes the particular meaning the disease acquires in a person’s life. According to Blumer [24], people act toward things according to what these things mean to them, meanings that arise from social interaction [24].

Illness experiences among Chilean women with SS have been previously described, showing that experiences cluster around common features, including limitations, pain, difficulties, coping, and attitudes toward treatment. However, these findings resulted from a cluster analysis whereby illness experiences were prompted by the researchers [25].

In this research, the concept of illness experience is defined according to a phenomenological approach: as a cognitive–emotional and behavioral response as a result of the interpretation of illness phenomena [26]. An approach to understanding the experience of diseases based on exploring patients’ interpretations is an important link between medical science, clinical practice, and the experiences of patients and health professionals [27]. Exploring disease experiences allows us to understand aspects not recognized by the classic health models, which can be fundamental to helping the patient achieve not only a better state of health but also a more satisfactory life.

Although several models explain aspects of health behaviors that are useful in certain interventions, human experience goes beyond predefined models. As such, research from a comprehensive perspective can deliver a more comprehensive outlook, thus providing elements targeting patient-centered therapies, improving the quality of health care, and providing better results in the quality of life of people affected by the disease.

Conceptual models of HRQoL and OHRQoL have been developed to link the multiple disease-affected domains of patients’ lives in an attempt to make sense of them. However, it is not possible to use such scales to explore the extent and complexity of the impact of symptoms on individuals, and there are limitations as to how such models can provide insight into patient perceptions [28].

The purpose of this qualitative research was to understand the disease experience among women suffering from SS from the patient’s subjective point of view to understand its impact on their overall well-being. The question that guided this research was, “What illness experiences determine well-being and a satisfactory life at an individual and social level?”

## 2. Materials and Methods

### 2.1. Design and Sample

The research had an analytical–relational design and was qualitative and cross-sectional. It was based on the approach and procedures proposed by Grounded Theory [29], using in-depth, semi-structured interviews.

Participants were recruited via the treating doctors at the University of Chile Clinical Hospital and a Facebook message by the National Association of Sjögren Patients. Interested participants could send an email to register. The participants were contacted by telephone to present the project details and carry out the interview, after which a date for a face-to-face meeting was agreed upon.

The inclusion criteria comprised women aged 18 to 70 with a confirmed diagnosis of Sjögren’s syndrome (SS) by a rheumatologist from the University of Chile Clinical Hospital, adhering to the 2016 ACR-EULAR classification criteria [30]. Exclusion criteria included pregnancy, untreated chronic conditions, inpatient status, mental health disorders, and patients in the acute phase of SS. A total of 15 women aged between 29 and 68, all with confirmed SS diagnoses, were included, without differentiation between primary or secondary SS. These women exhibited a range of symptoms and glandular or systemic manifestations of the disease.

The sampling method employed was theoretical [29], signifying that participant selection and data collection decisions were made concurrently, with data being collected, coded, and analyzed progressively without predefined criteria. Adjustments to the selection parameters were based on emerging findings to facilitate the development of a theoretical model rather than being predetermined [20,29].

### 2.2. Method of Information Production and Analysis

This study employed in-depth, semi-structured interviews as the method for generating information. In-depth interviews were selected for their ability to allow the respondents to articulate their perspectives on specific issues or experiences [31]. An interview guide was developed in line with this study’s objectives, which included identifying patients’ experiences related to themselves, significant others, the healthcare team, and the institution, as well as exploring experiences arising from the characteristics of the disease. The interview guide underwent a pilot test with a patient not included in the sample. Each interview, lasting approximately ninety minutes, was digitally recorded and transcribed verbatim, capturing the dialogue between the interviewer and interviewee in its entirety. The interviews were conducted by two members of the research team: a female psychologist (A.H.) and a male dentist and psychologist (G.R-A.), both with extensive experience in qualitative research. These interviews were conducted in a private room within the hospital setting.

The information collected was structured according to the specific objectives while respecting the narratives of the participants and, thus, describing their experiences from their point of view, in their language, and with their own expressions. Analysis criteria proposed by the Grounded Theory were used for the information analysis: open; axial; and selective analysis [29]. ATLAS.ti v.22 software was used to analyze the qualitative data. By applying this analysis procedure, the content evident in the conducted interviews could be described.

The information saturation point was reached after 15 interviews. This means that additional interviews do not foster any genuinely new understanding, as only similar cases are being seen [26]; as a result, the researcher acquires empirical confidence that a category has been saturated.

Information triangulation was carried out using the analysis from different researchers. This was conducted in three stages. First, two members of the research team independently analyzed all the information from the interviews. Next, the two researchers carried out a sharing session, where they performed the open, axial, and selective coding of the information. Finally, these were discussed and validated with the rest of the researchers. The study quality, meaning the reliability and authenticity, were protected by including these aspects. Additionally, the results were presented to one of the interviewed patients, who verified the categories established and the relational analyses performed. All the transcripts were sent to the participants by email; however, no feedback was received.

### 2.3. Ethical Considerations

The research was approved by the Ethics Committee of the Dentistry Faculty as well as the Ethics Committee of the Clinical Hospital (OAIC 896/17). The confidentiality of the participant information was protected by keeping the interview private and by protecting the information in databases restricted only to the research team. In addition, the anonymity of the participants was protected, both in the records and in the presentation and dissemination of information to other people. All participants provided written informed consent.

The participants did not receive any economic benefits from this study except for the transport expenses to the place of the interview.

## 3. Results

**Figure 1:**
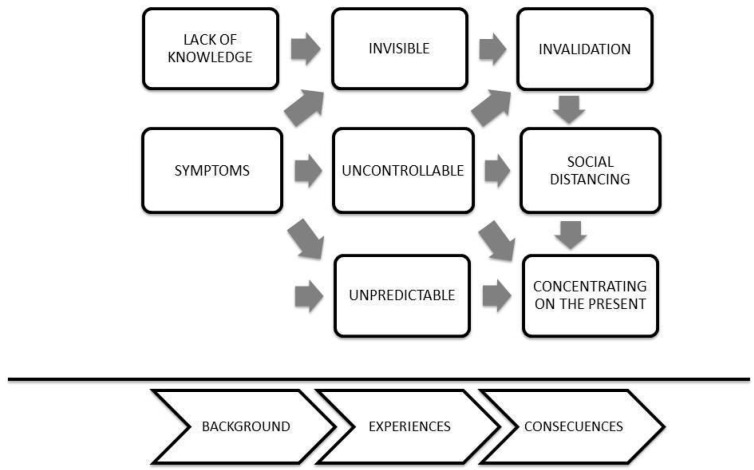
Central aspects of the disease experience.

The analysis enabled the identification of three central aspects of the disease experience, which were identified as invisible, uncontrollable, and unpredictable (Figure 1). All of them are intertwined, allowing us to understand the experiences of those suffering from SS.

1. Invisible

SS is experienced as a physically invisible disease. The organ deterioration caused by the disease is not observable to the naked eye, does not show any obvious effects, and is imperceptible to others. Discomfort, such as fatigue, dryness in all its presentations (eye, mouth, vaginal), and body pain, are experienced internally and are imperceptible to others.

*The disease is sort of silent, and also, it is not something that can be seen, like external*

*(PA: 8: 578)*

*You look good because ultimately you are not missing a hand or anything, you are complete, but you feel awful, meaning the process is going on inside*

*(SA:9:1012)*

*The symptoms it presents are not so disabling as to worry about them*

*(VV: 5: 893)*

As the symptoms are invisible, it is difficult for others to notice them, and they are rarely classified as severe or disabling, which means that those who suffer from this disease are attributed characteristics such as laziness, demonstrating disinterest, and lacking drive; this affects sufferers at the personal and professional levels.

The disease’s invisibility leads to an invalidation of the symptoms and a lack of empathy from those who should form the patient’s support network, which weakens social support. On the contrary, there is a social devaluation because since the disease does not present visible signs, the patient’s situation is considered an exaggeration or an overreaction to any physical discomfort.

*People think you are lazy, you know?*

*(SA: 9:229)*

*I don’t get tired because I want to get tired or because I am old, I get tired because I have this thing*

*(IV: 3: 347)*

*I mean, I am doing something, and I have to go lie down because I can’t anymore, I have no strength and everyone tells me, ‘you are so lazy!’*

*(IV: 3:581)*

This leads to a social distancing, typified in some cases by the avoidance of socializing opportunities (meetings, parties, dinners, etc.) but, above all, represented by silence; this means that patients avoid talking about their symptoms and pains; they avoid seeking help since they feel misunderstood and prefer not to verbalize what is happening to them, arguing that others will not understand and that they do not want to be stigmatized as exaggerators or lazy.

*I don’t try to explain it to anyone, nor have I told anyone, because I find it too difficult to get someone to understand me*

*(VV: 5: 455)*

*I just hang out with people my age (young) and I think it is very difficult for someone to understand me, to understand that you might be tired and fatigued… in fact, my husband sometimes tells me “You were born tired! And I respond, Yes!, I take it with humor. I understand them because they have never felt this, so I say: well, how can I begin to explain it to them!*

*(VV: 5:461)*

2. Uncontrollable

The uncontrollability is related to the symptoms that are typical of SS; it specifically refers to pain and fatigue, both difficult to control in their onset and evolution.

Although one of the main symptoms of SS is dry mucous membranes, the interviewees have multiple strategies to control it, such as always having chewing gum and water for dry mouth, using artificial tears for dry eyes, and using lubricants for vaginal dryness. In this way, this symptom can be managed correctly by the patients.

On the other hand, most interviewees report having no control over the emergence of symptoms such as pain and fatigue. They understood that once these symptoms appear, there are not many alternatives for combating and reducing them, so one strategy used is to focus on positive emotions, trying to cultivate a better mood, as this would help decrease the intensity of the pain experienced.

*There are days when I wake up with a lot of pain, and there is nothing that relieves my pain*

*(YE: 15: 420)*

*There are days that I have no motivation for anything, and it is not that I have a reason, like: ah I am sad about this! There’s no reason and that is the worst of all*

*(YE: 15: 124)*

Although the interviewees do not establish a relation of causality between their mood and the pain experienced, they recognized that it has an impact on the disease, so an indirect way of dealing with it is to propose being substantially emotionally independent from the difficulties experienced; this means that they try to be resilient and have a positive view of life.

*My pains went down a lot because I was not stressed*

*(MO: 2:1298)*

*I have noticed that in periods of stress, after the stress passes, like, the crisis comes to me*

*(YE15: 106)*

*External agents, which are work, family, worries, and problems, make you fall faster, you get more fragile when faced with the illness*

*(PD17: 149)*

This uncontrollability of the pain and fatigue symptoms results in social distancing—an element also present in the invisibility of SS—and focusing on the present. Social distancing is explained by the fact that the pain and fatigue mean that patients need to rest and suspend their activities, which, in many cases, translates into peacefulness but also loneliness.

In addition, uncontrollability in terms of the emergence and evolution of certain symptoms means that SS sufferers only worry about the present because the pain and fatigue force them to modify their plans often; as a result, planning further than every day is avoided, and possible activities are evaluated on a day-to-day basis. This necessarily implies flexibility for modifying their plans and rescheduling activities, even if they include other people or involve work.

*You plan to do four activities and you finish one if you’re lucky. And then you end up tired, and you look and say, darn it, I have done nothing and I’m so tired, it is horrible*

*(CM: 11: 953)*

*I intend to continue working, always work, for this not affect my work, I don’t think much about what can happen in the future*

*(PB: 1: 1799)*

3. Unpredictable

Unpredictability is closely related to uncontrollability. The interviewees indicated that they cannot predict the emergence or intensity of pain and fatigue symptoms. This reinforces their focus on the present, which is also observed in the uncontrollable experience of the disease. This translates into concentrating on the present, living day to day, and not planning for the future.

It is worth mentioning that this experience is mainly related to the pain and fatigue symptoms, which end up being more complex for patients to handle since symptoms like dryness—always being present and being a constant—allow for the adaptation and development of stable coping strategies over time.

By contrast, pain and fatigue, being unpredictable and uncontrollable, prevent the generation of effective coping strategies for dealing with the disease, which has repercussions on the quality of life and the vision for the future of SS sufferers.

*There are days when I wake up really well and out of nowhere, I feel bad*

*(YE:15:116)*

*There are days that I wake up very well and there are days that I don’t feel like doing anything, I get up wanting to go to bed, heavy body, pain in my hands, I don’t want to do anything or let anyone speak to me, but it’s not always, it’s sometimes*

*(YE:15:104)*

*You go with the pain, it is like putting on a t-shirt, a vest, a gown, I don’t know, you go all day with the pain*

*(CM: 11: 983)*

## 4. Discussion

Emotional balance is the most bothersome patient’s experience with Sjogren’s disease. Anxiety, depression, and emotional struggle are distinct experiences describing the emotional experiences of patients as they try to navigate life while managing a chronic, disruptive, and unpredictable illness [32].

The results of this study show that the experience of patients with SS is defined by three central phenomena: invisibility; uncontrollability; and unpredictability. These phenomena align with the characteristic features shared by other CDs, including silent progression and incurability, alternating symptomatic and asymptomatic episodes [18]. However, SS possesses unique attributes that set it apart, notably, the absence of visible organ damage, its low prevalence in the population, the peculiar etymology derived from the Swedish language (originating from a doctor’s surname rather than a clinical presentation), and the inadequate comprehension of the disease among physicians [33]. Furthermore, SS presents with invisible symptoms such as mucosal dryness syndrome, fatigue, and chronic body pain, eliciting a societal response characterized by skepticism [34].

The findings of this study are aligned with the general description of illness experience in chronic diseases, where symptoms are perceived as unpredictable and uncontrollable [35]. Rheumatic diseases describe the experience of invalidation, and we found that SS patients experience the phenomenon of invisibility. These two phenomena are close but have different meanings: invalidation refers to neglect of the patient’s illness, and invisibility refers to ignorance of the patient’s condition. We were able to conclude that invisibility led to invalidation.

The literature describes that invalidation and uncontrollability lead to social distancing [35]; in our study, this would explain the social incomprehension and discrimination experienced by SS patients.

The invisibility phenomenon comes from insufficient information given by health professionals, modifying the illness experience in patients and their social environment. [34]. The physician’s improper guidance intensifies the patient’s feeling of not knowing what is happening, leaving the patient disoriented and without reliable information sources; normally, they end up searching the web, which leads to more confusion [36].

The invalidation phenomenon is mainly a descriptor for rheumatologic diseases with high-level symptomatology but low-level visible damage to the body, such as fibromyalgia [37]. Fibromyalgia and SS have common characteristics, including chronic pain, fatigue symptoms, and the absence of socially visible signs of the disease. The invalidation includes non-acceptance of the disease by others, misunderstanding, disbelief, rejection, and stigmatization, which are experienced as denial, sermonizing, overprotection, lack of support, and ignorance about the patient’s condition [37].

In our study, the invalidation leads patients to feel constantly questioned and judged about their real condition as patients. Due to this, they tend to isolate themselves in order to avoid criticism, skepticism, and questioning.

Two dimensions of invalidation have been identified: lack of understanding; and discounting (belittling/underestimating) [38]. Both elements were identified in the patient experiences in this study. Lack of understanding implies non-recognition, non-understanding, and lack of emotional support for the person. Discounting represents the negative social response and social rejection, including disbelief, scolding, disregard for their inability to work, ignorance about the fluctuation of symptoms, and giving unhelpful advice [38]. It should be mentioned that invalidation depends both on the response of others to the person (objective invalidation) and the assessment that the person makes of those responses (subjective invalidation). The severity of the invalidation depends not only on the current invalidation from the social environment but also on the person’s perception and abilities [39].

Unlike invisibility and invalidation, uncontrollability and unpredictability are clearly described phenomena for other chronic diseases, mainly those diseases that are accompanied by chronic pain and appear not to be an SS phenomenon. In the case of the present study, both phenomena are directly related to fatigue and pain symptoms, which cannot be avoided or effectively treated. It is a subjective perception, therefore, since it is the sick person’s interpretation. That means that given the same disease situation, different people can perceive different degrees of control. However, uncontrollability and unpredictability arose as a hallmark characteristic of women interviewed in this study, so we propose that the condition of SS is not only marked by difficult symptoms of clinical control but also by a social situation of misunderstanding that intensifies the perception of lack of control; that is, there is an additive effect that does not contribute to the patient’s well-being. The perception of control over chronic disease symptoms is critical for the patient’s well-being [40] and has been described as modulating mood, stress, pain, and fatigue in patients with fibromyalgia [41]. There may be a vicious circle between the perception of control and the symptoms. Fatigue and body pain cause a perception of lack of control, and at the same time, this accentuates the fatigue and pain symptoms.

The lack of control and predictability are associated with a framework known as Learned Helplessness, which affects both psychological functions and physical symptoms. One of the situations most affected by the lack of control is the experience of body pain [42], and perception of control is suggested as an effective strategy for reducing the pain and disability it causes [43]. This is explained by the fact that a greater acceptance of pain has been associated with lesser attention to the pain [44], low levels of anxiety and avoidance, less depression, and fewer physical and psychosocial disabilities [45]. In turn, the acceptance of pain dampens the negative effects involved in pain [46].

Uncontrollability and unpredictability are not only experienced when the disease is diagnosed, but they are also greatly accentuated in the pre-diagnostic stage, where patients usually go through various doctors and other health professionals in search of a definitive diagnosis. The phenomena of uncontrollability and unpredictability of events have been described as a source of stress and anxiety, and they also accentuate somatic symptoms [47]. This explains why SS patients experience relief once the diagnosis of SS has been obtained because the uncertainty of not having an accurate diagnosis is over [48], and this gives them a certain degree of perception of control and prediction when focusing on one specific element, which was previously a broad diffuse spectrum.

The experience of a chronic disease typified by the presence of strong symptoms and the inability to perform daily tasks, together with the lack of visible organ damage and being poorly understood by the community, presents a number of psychosocial phenomena shared with other chronic diseases, but invisibility, invalidation, social distancing, and everyday improvisation are added, and in which the phenomena of uncontrollability and unpredictability stand out for the type of symptoms experienced.

Going from the invisible, uncontrollable, and unpredictable to a situation of being seen as sick and with recognized limitations, with clear information about their disease and therapeutic possibilities, along with emotional support from the health team and family environment, becomes a strategic objective for offering well-being and fostering a good life despite the complications of the disease. As a result, it is not only a medical intervention but, in fact, a systemic task, which includes the health team as well as the patient and their family environment.

## Data Availability

The raw data supporting the conclusions of this article will be made available by the authors on request.
